# Supplementary material for: PhoPQ-mediated lipopolysaccharide modification governs intrinsic resistance to tetracycline and glycylcycline antibiotics in Escherichia coli
Source: mSystems. 2024 Sep 30;9(10):e00964-24. doi: 10.1128/msystems.00964-24 (PMC11495068; doi:10.1128/msystems.00964-24)
Supplement: Supplemental material — Supplemental figures and tables. [file msystems.00964-24-s0001.docx]

***Supplementary materials***

- 1. **Supplementary Figures**

**
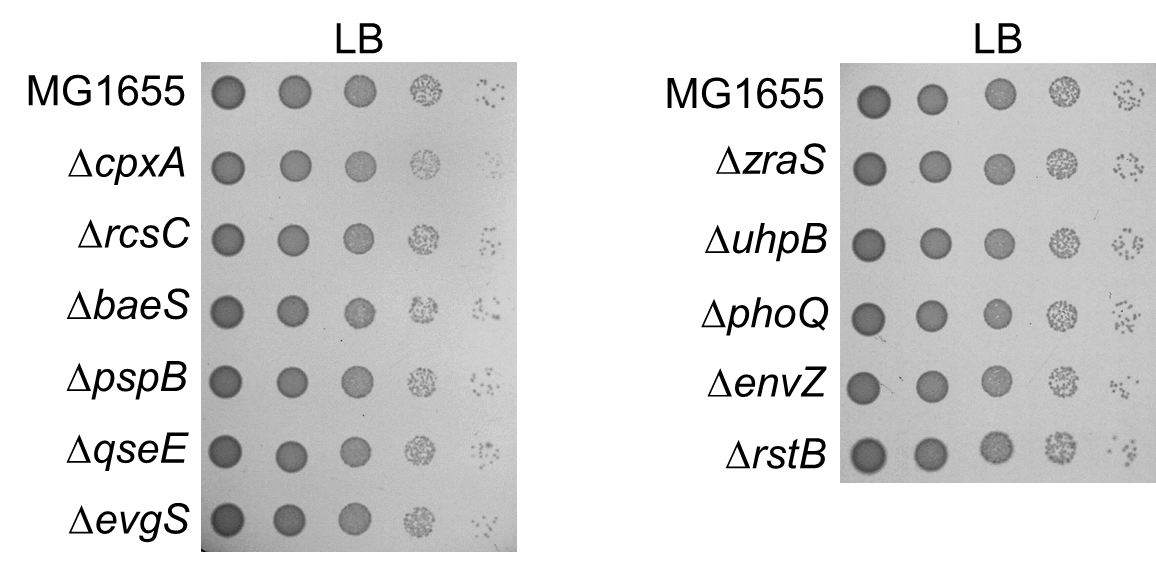
**

**FIG S1** The effect of inactivation of two-component system sensor kinases on cell growth in LB medium. The cells of the indicated strains were serially diluted from 10^8^ to 10^4^ cells/ml in 10-fold steps and spotted onto LB plates

**
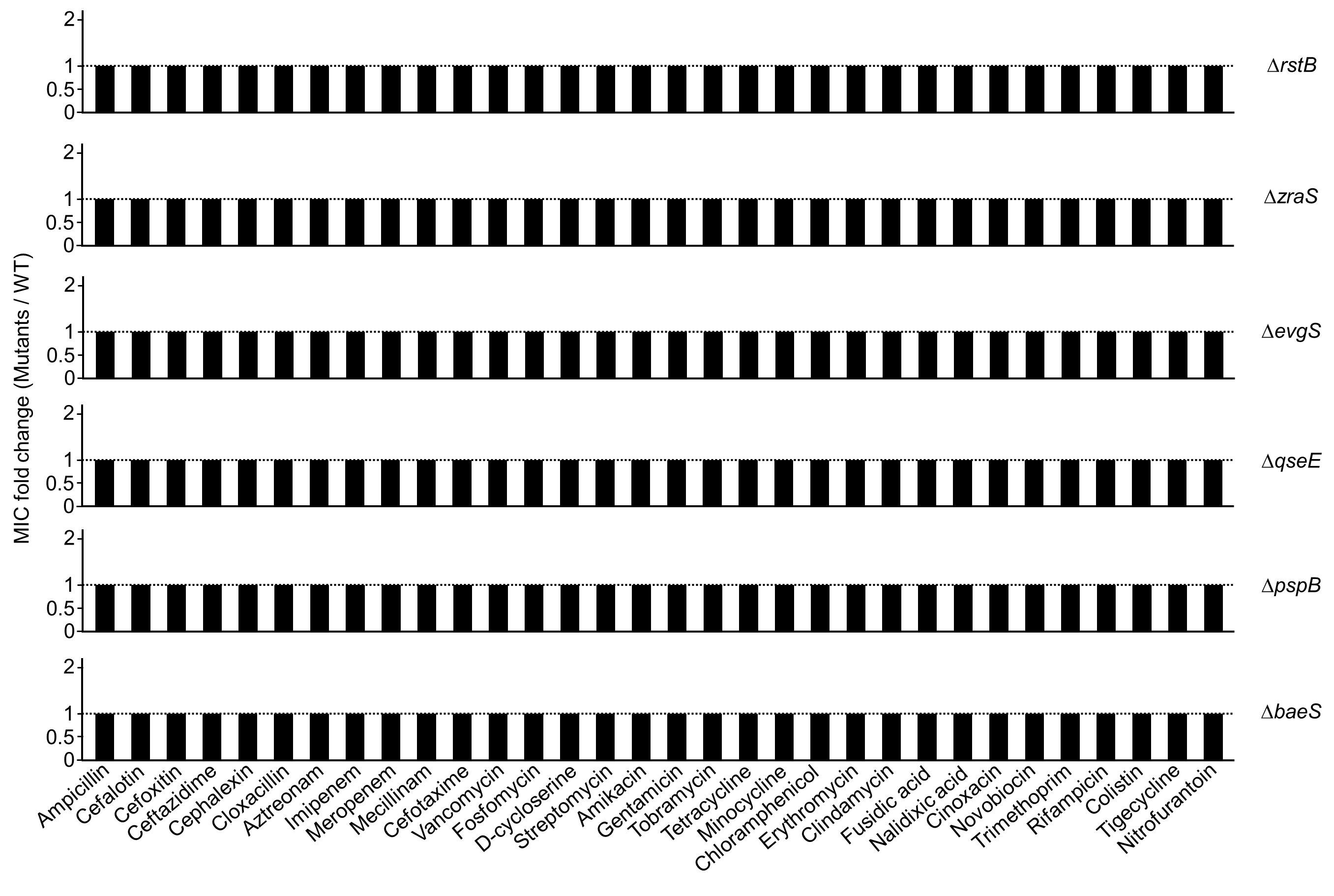
**

**FIG S2** The effect of inactivation of two-component system sensor kinases on intrinsic antibiotic resistance. The MICs of various antibiotics were measured against the wild-type and indicated mutant strains in MH medium. The relative MIC values for the indicated mutant cells compared to those for the wild-type cells are presented.

**
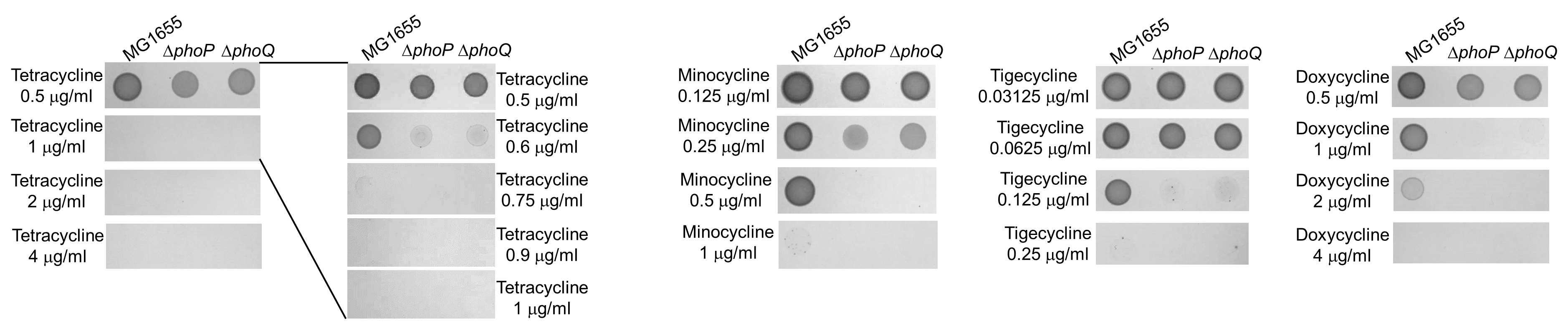
**

**FIG S3** Increased susceptibilities of the Δ*phoP* or Δ*phoQ* mutant to tetracycline and glycylcycline antibiotics. The MICs of indicated antibiotics were measured against the wild-type and Δ*phoP* or Δ*phoQ* mutant strains in MH medium. The experiments were performed in triplicate, and a representative image is presented.

**
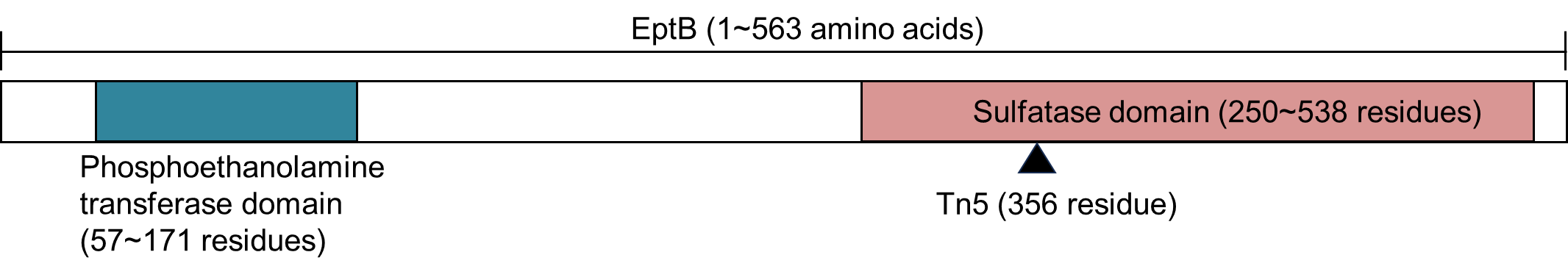
FIG S4** Schematic representation of a Tn5 insertion site. The Tn5 insertion site of suppressor mutant is indicated using a black arrow.

**
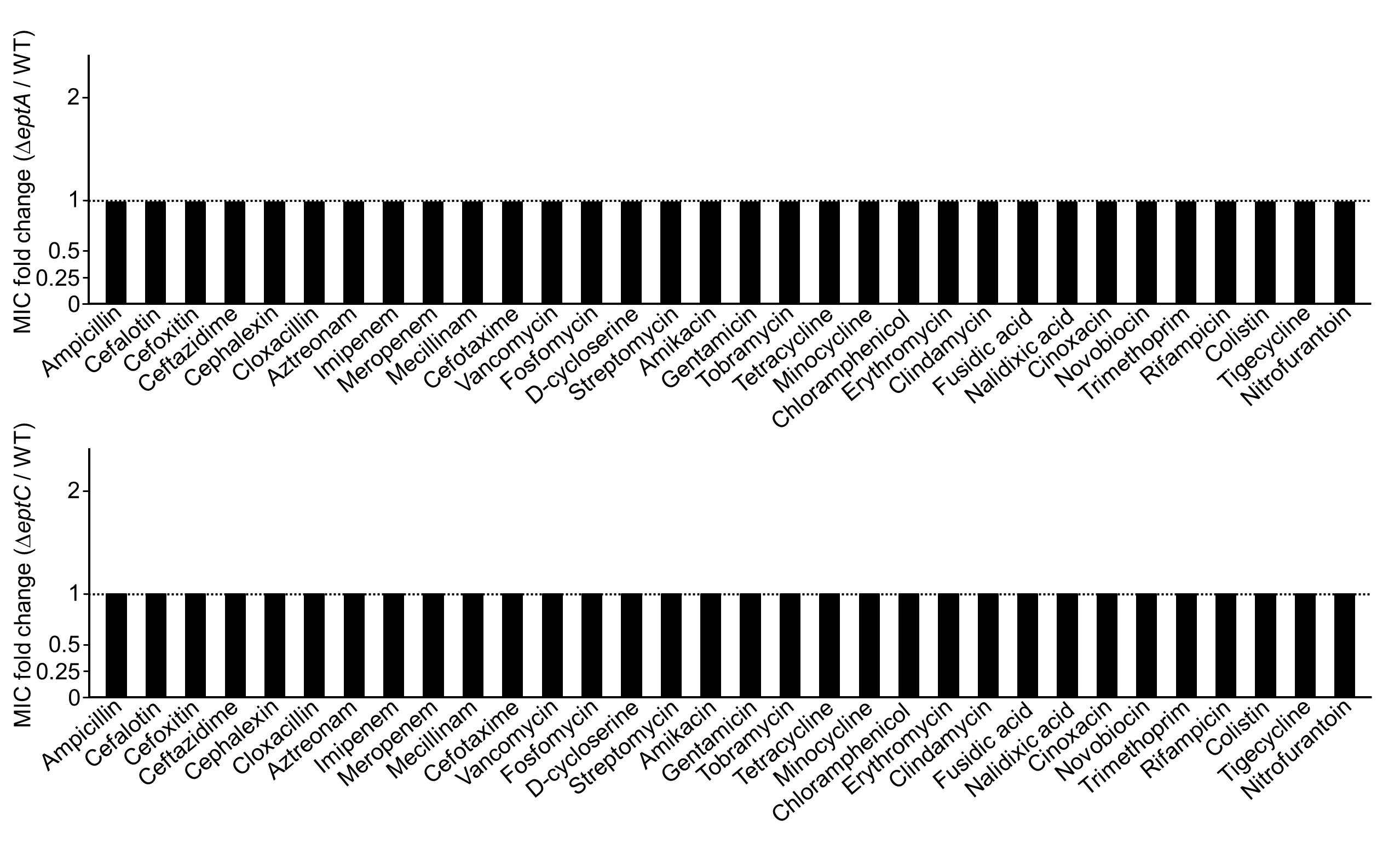
**

**FIG S5** The effect of EptA or EptC depletion on the MICs of antibiotics. The MICs of various antibiotics were measured against the wild-type and Δ*eptA* or Δ*eptC* mutant strains in MH medium. The relative MIC values for the Δ*eptA* or Δ*eptC* mutant cells compared to those for the wild-type cells are presented.

- 1. **Supplementary Tables**

**Supplementary Table S1.** ***Escherichia coli* strains and plasmids used in this study.**

| Strain or plasmid | Genotype or phenotype | Source or Reference |
| --- | --- | --- |
| **Strains** |  |  |
| MG1655 | F^-^ λ^-^ *ilvG*^-^ *rfb*-50 *rph*-1. Wild type *E. coli* K-12 | (1) |
| MG1655 Δ*cpxA* | MG1655 *cpxA::frt* | This study |
| MG1655 Δ*rcsC* | MG1655 *rcsC::frt* | This study |
| MG1655 Δ*baeS* | MG1655 *baeS::frt* | This study |
| MG1655 Δ*pspB* | MG1655 *pspB::frt* | This study |
| MG1655 Δ*qseE* | MG1655 *qseE::frt* | This study |
| MG1655 Δ*evgS* | MG1655 *evgS::frt* | This study |
| MG1655 Δ*zraS* | MG1655 *zraS::frt* | This study |
| MG1655 Δ*uhpB* | MG1655 *uhpB::frt* | This study |
| MG1655 Δ*phoQ* | MG1655 *phoQ::frt* | This study |
| MG1655 Δ*envZ* | MG1655 *envZ::frt* | This study |
| MG1655 Δ*rstB* | MG1655 *rstB::frt* | This study |
| MG1655 Δ*phoP* | MG1655 *phoP::frt* | This study |
| MG1655 Δ*mgtA* | MG1655 *mgtA::frt* | This study |
| MG1655 Δ*corA* | MG1655 *corA::frt* | This study |
| MG1655 Δ*eptA* | MG1655 *eptA::frt* | This study |
| MG1655 Δ*eptB* | MG1655 *eptB::frt* | This study |
| MG1655 Δ*eptC* | MG1655 *eptC::frt* | This study |
| MG1655 Δ*phoP* Δ*eptA* | MG1655 *phoP::frt eptA::frt* | This study |
| MG1655 Δ*phoP* Δ*eptB* | MG1655 *phoP::frt eptB::frt* | This study |
| MG1655 Δ*phoP* Δ*eptC* | MG1655 *phoP::frt eptC::frt* | This study |
| **Plasmids** |  |  |
| pACYC184 | A low copy number cloning vector; Cm^R^, Tet^R^ | (2) |
| pACYC-PhoP | pACYC184-based expression vector for PhoP, Cm^R^ | This study |
| pBAD24 | Expression vector under control of arabinose-inducible promoter, Amp^R^ | Addgene |
| pBAD24(Cm) | pBAD24-based expression vector, Cm^R^ | (3) |
| pBAD-MgtA | pBAD24(Cm)-based expression vector for MgtA, Cm^R^ | This study |
| pBAD-CorA | pBAD24(Cm)-based expression vector for CorA, Cm^R^ | This study |
| pBAD-OmpT | pBAD24(Cm)-based expression vector for OmpT, Cm^R^ | This study |
| pBAD-BorD | pBAD24(Cm)-based expression vector for BorD, Cm^R^ | This study |
| pBAD-PagP | pBAD24(Cm)-based expression vector for PagP, Cm^R^ | This study |
| pBAD-TolC | pBAD24(Cm)-based expression vector for TolC, Cm^R^ | This study |
| pBAD-FadL | pBAD24(Cm)-based expression vector for FadL, Cm^R^ | This study |
| pBAD-EptB | pBAD24(Cm)-based expression vector for EptB, Cm^R^ | This study |
| pKD13 | Template plasmid for the amplification of the kanamycin-resistance gene bordered by FRT sites, Km^R^ | (4) |
| pKD46 | λ Red recombinase expression plasmid Ts replicon, Amp^R^ | (4) |
| pCP20 | FLP helper plasmid Ts replicon, Amp^R^, Cm^R^ | (4) |
| pRL27 | Tn5-RL27 (Km^R^-*ori*R6 K) delivery vector: circularized PCR fragment from pRL23 (primers *tet*Ap-for and *oriT-rev*) | (5) |

**Supplementary Table S2.** **Oligonucleotides used in this study**

| **Name** | **Oligonucleotide sequence (5’–3’)** | **Use(s)** |
| --- | --- | --- |
| CpxA-FRT-F | ATGATAGGCAGCTTAACCGCGCGCATCTTCGCCATCTTCTGGCTGACGCTGTGTAGGCTGGAGCTGCTTC | Deletion |
| CpxA-FRT-R | CACATTAAATCGTTGGGCGGATCGTTCGCCAGCTCCGCTTCGACATGCTGATTCCGGGGATCCGTCGACC |  |
| RcsC-FRT-F | CGCTACCTTAACCACACTCCATCGGTCACCTGAGGCGGAGCTTCGCCCCTGTGTAGGCTGGAGCTGCTTC |  |
| RcsC-FRT-R | CTGACGAATTTCCGATTCTCGCTGATGTAACGCATTAACGATGTAAAAAAATTCCGGGGATCCGTCGACC |  |
| BaeS-FRT-F | ATGAAGTTCTGGCGACCCGGTATTACCGGCAAACTGTTTCTGGCGATTTTGTGTAGGCTGGAGCTGCTTC |  |
| BaeS-FRT-R | TTGCCATGCTGCGCATACTGCTCGCCAAGCGCATCACTTAACAGTTGTAAATTCCGGGGATCCGTCGACC |  |
| PspB-FRT-F | CATCCAGGCGGCGTCCGAACGCGCCGCCGCTCATCGTCTAAGGAGTACTTGTGTAGGCTGGAGCTGCTTC |  |
| PspB-FRT-R | CACTGCGACCAGAACGATTGCTGTAATGCAGCCATAACCAGATCGGTAAAATTCCGGGGATCCGTCGACC |  |
| QseE-FRT-F | ACTTCCCCTCGGTTAGCATCAGGCTATTCGCGTCTGACGAGAGTAACACCGTGTAGGCTGGAGCTGCTTC |  |
| QseE-FRT-R | AGTGCGGTTAACCAGCGCCGCCTGATCGCTAAGCGCATTCAGGCTTTGCCATTCCGGGGATCCGTCGACC |  |
| EvgS-FRT-F | ATGAAGTTTTTACCCTATATTTTTCTTCTCTGTTGTGGTCTTTGGTCGACGTGTAGGCTGGAGCTGCTTC |  |
| EvgS-FRT-R | GTTTGGGACTTATGTACTGCAATCACAAGATTTTTTTTGCTCGCTAACCAATTCCGGGGATCCGTCGACC |  |
| ZraS-FRT-F | AGATGGCATGATTTCTGCTGTCAGAAAGGGATGAGCAGGCAAAGAAGAAGGTGTAGGCTGGAGCTGCTTC |  |
| ZraS-FRT-R | CAGTAATGCCTGGCGGTCTGCCTCGCTTGCCCGCCCATAATCACGAATCAATTCCGGGGATCCGTCGACC |  |
| UhpB-FRT-F | ATGAAGACGTTGTTCTCCCGCTTAATTACCGTTATTGCCTGCTTTTTTATGTGTAGGCTGGAGCTGCTTC |  |
| UhpB-FRT-R | TCCGCGCCCAGCAATACGGGCCAGTATCCGCGCGGGCATTGCAGCATTAGATTCCGGGGATCCGTCGACC |  |
| PhoQ-FRT-F | ATGAAAAAATTACTGCGTCTTTTTTTCCCGCTCTCGCTGCGGGTACGTTTGTGTAGGCTGGAGCTGCTTC |  |
| PhoQ-FRT-R | TTTTCCCACTTCGCAAGGGTATAGAACAGATTGCTCTCGCCACGTAACAGATTCCGGGGATCCGTCGACC |  |
| EnvZ-FRT-F | ATGAGGCGATTGCGCTTCTCGCCACGAAGTTCATTTGCCCGTACGTTATTGTGTAGGCTGGAGCTGCTTC |  |
| EnvZ-FRT-R | CCGTCCTCCAGTTGCAGTTTGTCGGTCATCAACATACGCACTTCGTACGCATTCCGGGGATCCGTCGACC |  |
| RstB-FRT-F | ATGAAAAAACTGTTTATCCAGTTTTACCTGTTATTGTTTGTCTGCTTCCTGTGTAGGCTGGAGCTGCTTC |  |
| RstB-FRT-R | TTCAGAGTTTTACCCCAGTCGTGTGGGGGGATCTCACGCAATTCGCTGCGATTCCGGGGATCCGTCGACC |  |
| PhoP-FRT-F | CACATAATCGCGTTACACTATTTTAATAATTAAGACAGGGAGAAATAAAAGTGTAGGCTGGAGCTGCTTC |  |
| PhoP-FRT-R | AGTCACATAATCATCAGCACCGGCACTTAATACTTCGACTTTGTCCTGCCATTCCGGGGATCCGTCGACC |  |
| MgtA-FRT-F | ACGCCTGAAGACATTTCTGTACTGTTTCAGACAGTGCGGAGGGACTCCTTGTGTAGGCTGGAGCTGCTTC |  |
| MgtA-FRT-R | GCTTCCCACCAGTCGCCTTTGGTGTAACCATTGATTAACAGCACCACCGGATTCCGGGGATCCGTCGACC |  |
| CorA-FRT-F | CTGTGTGCCCACCGAACTGTCCGATATTTTAAGCATTGGGAGTCCCGGTCGTGTAGGCTGGAGCTGCTTC |  |
| CorA-FRT-R | ACAGGTGGCTTAGCCAGACTAAGCCACCGCTCTCGTTTTTTACAACCAGTATTCCGGGGATCCGTCGACC |  |
| EptA-FRT-F | TAATTTTGCTTTGCGAGCATATGCGCACTTTGTTCGATGGAAACACCGTGGTGTAGGCTGGAGCTGCTTC |  |
| EptA-FRT-R | GTTCTGGTGAGGCACGCGGTCGCAGGCACCTTTACAGCCGCCATCGTTGTATTCCGGGGATCCGTCGACC |  |
| EptB-FRT-F | AAAAGCCACTAAGCAGGGTGTTATCACCTGTTTGTCCAGGGTTTGTTTGCGTGTAGGCTGGAGCTGCTTC |  |
| EptB-FRT-R | GTCCGCCATCGTGTTGCTGTAGAACCACATTTCGCTCTGCATAGCGTAGAATTCCGGGGATCCGTCGACC |  |
| EptC-FRT-F | AAGGAATTGTCGTTACATTCGGCGATATTTTTTCAAGACAGGTTCTTACTGTGTAGGCTGGAGCTGCTTC |  |
| EptC-FRT-R | ATCCAGAAGGTTTTATAACCCGCCTGTTTCATCATGTTCATCAGCGACGGATTCCGGGGATCCGTCGACC |  |
| CpxA-cfm-F | TGGTCGCGGCTATCTGATGGTTTCTGCTTC | Deletion confirm |
| CpxA-cfm-R | TTATCAATCGCCCGGAACAGACGCCGCCAC |  |
| RcsC-cfm-F | AGAGAGAACATTGCGGTAACACGCTTTTAC |  |
| RcsC-cfm-R | CTGAGCCTGATCGGAACTCAGATTAAATTC |  |
| BaeS-cfm-F | AGCTATTTCGCGGCGAAAAAGGAGCGCGCA |  |
| BaeS-cfm-R | AAGCGATCATTGTTGCGCAGGAAGCGCCAG |  |
| PspB-cfm-F | AGCCGATGATGCAATCAGCGAACAACTGGC |  |
| PspB-cfm-R | GCCAGTTGCGCTAATCGCTGCTGCTCACTT |  |
| QseE-cfm-F | CCGGGTAATTCCCGGCTTTGTTGTATCTGA |  |
| QseE-cfm-R | CGCTTCACTGCGCCGGGCATCAATAAGCGT |  |
| EvgS-cfm-F | TCGCACAACGTAACAAAATCGGCTAACCAC |  |
| EvgS-cfm-R | TGCTGCGAATCGGTATGCAACAACGTAGCC |  |
| ZraS-cfm-F | GTGTTACAGCGCAGGGTAAGCGCTGATAAA |  |
| ZraS-cfm-R | CAGAGCGCGGATAAGCACATTACCTTTTTC |  |
| UhpB-cfm-F | GAAAAACTGGGCGTCAGTAACGACGTAGAG |  |
| UhpB-cfm-R | TCAAACCTTAGACATAGCGTTGAGGTAGAG |  |
| PhoQ-cfm-F | CGGCCAGGGCTATCTGTTCGAATTGCGCTG |  |
| PhoQ-cfm-R | AGCGTCATGGTGGGGCTTTGCTTGTCGATA |  |
| EnvZ-cfm-F | CTACGTCTTTGTACCGGACGGCTCTAAAGC |  |
| EnvZ-cfm-R | GAGATCCCCAGCTCACGGTAGATCTCCCGA |  |
| RstB-cfm-F | CTTTTTGCGCCTCATGCATGGGAATAAGCG |  |
| RstB-cfm-R | TACTCAGTGGCTCGACACGCAGATCGAAAG |  |
| PhoP-cfm-F | TGTCCGGCCTGCTTATTAAGATTATCCGCT |  |
| PhoP-cfm-R | CGCCATCACCTCTTCAATATGAAACGGTTT |  |
| MgtA-cfm-F | AGCTTTATTACCTTCAGGTAAGGCTTCGCC |  |
| MgtA-cfm-R | CGTCGAGGTAACAATCATCGGCAACATTTC |  |
| CorA-cfm-F | CTGAGTCAGGCTGTTTAATGGTCTGAAACC |  |
| CorA-cfm-R | AAGAGGTAAAAGGGTTAAATACCTGGAGCC |  |
| EptA-cfm-F | CCCTTAATCCAGCAAACATAAAAGC CAACC |  |
| EptA-cfm-R | CGCCGTTGATGCACTGATCAGGTAGATTCA |  |
| EptB-cfm-F | CTTATCTGACTACCTCCGCACTTTTTCCCT |  |
| EptB-cfm-R | CGCACCAATCTGCTCACGATAAGCAATGTT |  |
| EptC-cfm-F | TTCTGAAATACTTCTGTTCTAACACCCTCG |  |
| EptC-cfm-R | CTGGTTCATGTAGTACTGCTTGTCGGTCTG |  |
| pACYC184-PhoP-F | CCCGTCCTGTGGATCCAAACCTCGTATCAGTGCCGG | pACYC184 cloning |
| pACYC184-PhoP-R | CCCAGCGCGTCGGCCGGACGCAGTAATTTTTTCATC |  |
| pBAD24-MgtA-F | CTAGCAGGAGGAATTCATGTTTAAAGAAATTTTTAC | pBAD24 cloning |
| pBAD24-MgtA-R | GCAGGTCGACTCTAGATTATTGCCAGCCGTAAC GAC |  |
| pBAD24-CorA-F | CTAGCAGGAGGAATTCATGCTGAGCGCATTTCAACT |  |
| pBAD24-CorA-R | GCAGGTCGACTCTAGAAGACTAAGCCACCGCTCTCG |  |
| pBAD24-OmpT-F | CTAGCAGGAGGAATTCATGCGGGCGAAACTTCTGGG |  |
| pBAD24-OmpT-R | GCAGGTCGACTCTAGAGTTGGCGTTCTTAAAATGTG |  |
| pBAD24-BorD-F | CTAGCAGGAGGAATTCATGAAAAAAATGCTACTCGC |  |
| pBAD24-BorD-R | GCAGGTCGACTCTAGAATGCAATTATTTTGAGCAAT |  |
| pBAD24-PagP-F | CTAGCAGGAGGAATTCATGAACGTGAGTAAATATGT |  |
| pBAD24-PagP-R | GCAGGTCGACTCTAGATCATTTGTCTCAAAACTGAA |  |
| pBAD24-TolC-F | CTAGCAGGAGGAATTCATGAAGAAATTGCTCCCCAT |  |
| pBAD24-TolC-R | GCAGGTCGACTCTAGACGTCGTCATCAGTTACGGAA |  |
| pBAD24-FadL-R | CTAGCAGGAGGAATTCATGAGCCAGAAAACCCTGTT |  |
| pBAD24-FadL-R | GCAGGTCGACTCTAGACTATGCAGGTGACTTTATCC |  |
| pBAD24-EptB-F | CTAGCAGGAGGAATTCATGAGATACATCAAATCGAT |  |
| pBAD24-EptB-R | GCAGGTCGACTCTAGATTAGTTAGCCGCTGCCTCTT |  |
| pRL27-cfm-F1 | GGTTGTAACACTGGCAGAGCATTACG | Random mutagenesis confirm |
| pRL27-cfm-F2 | CAACGCAGACCGTTCCGTGGCAAAGC |  |
| pRL27-cfm-R1 | TCTCATGTTTCACGTACTAAGCTCTC |  |
| pRL27-cfm-R2 | ATCAGCAACTTAAATAGCCTCTAAGG |  |
| RT-16s rRNA-F | AAATTGAAGAGTTTGATCATGGCTCAGATT | qRT-PCR |
| RT-16s rRNA-R | AATGAGCAAAGGTATTAACTTTACTCCCTT |  |
| RT-PhoP-F | ATGCGCGTACTGGTTGTTGAAGACAATGCG |  |
| RT-PhoP-R | TTCACGGCGAGAGAGATCAACCTGAAACGG |  |
| RT-EptB-F | TCAAATCGATTACACAGCAGAAGCTGAGCT |  |
| RT-EptB-F | TGATGCCATAACCAATGACCACATTAAGGA |  |

**References**

1. Blattner FR, Plunkett G, 3rd, Bloch CA, Perna NT, Burland V, Riley M, Collado-Vides J, Glasner JD, Rode CK, Mayhew GF, Gregor J, Davis NW, Kirkpatrick HA, Goeden MA, Rose DJ, Mau B, Shao Y. 1997. The complete genome sequence of *Escherichia coli* K-12. Science **277**:1453-1462.

2. Schottel JL, Bibb MJ, Cohen SN. 1981. Cloning and expression in *Streptomyces lividans* of antibiotic resistance genes derived from *Escherichia coli*. J Bacteriol **146**:360-368. <https://doi.org/10.1128/jb.146.1.360-368.1981>.

3. Park SH, Kim YJ, Lee HB, Seok YJ, Lee CR. 2020. Genetic Evidence for Distinct Functions of Peptidoglycan Endopeptidases in Escherichia coli. Front Microbiol **11**:565767. <https://doi.org/10.3389/fmicb.2020.565767>.

4. Datsenko KA, Wanner BL. 2000. One-step inactivation of chromosomal genes in *Escherichia coli* K-12 using PCR products. Proc Natl Acad Sci USA **97**:6640-6645. <https://doi.org/10.1073/pnas.120163297>.

5. Larsen RA, Wilson MM, Guss AM, Metcalf WW. 2002. Genetic analysis of pigment biosynthesis in *Xanthobacter autotrophicus* Py2 using a new, highly efficient transposon mutagenesis system that is functional in a wide variety of bacteria. Arch Microbiol **178**:193-201. <https://doi.org/10.1007/s00203-002-0442-2>.
